# Supplementary material for: Delays in seeking treatment for fever in children under five years of age in Nigeria: Evidence from the National Demographic Health Survey
Source: PLoS One. 2023 Dec 20;18(12):e0295772. doi: 10.1371/journal.pone.0295772 (PMC10732371; doi:10.1371/journal.pone.0295772)
Supplement: Attachment — Submitted filename: report Dr LOUGUE.pdf [file pone.0295772.s001.pdf]

Delays in seeking treatment for fever in children under five years of age in Nigeria: Evidence from the National Demographic Health Survey

General comments:

The topic is very interesting and the study provided very good insights improving treatment of under five children.

Corrections:

- In some comments, the author mistakenly commented as if information was about the mothers, example "However, mothers who had an unwanted pregnancy had a higher odds of delaying treatment for childhood fever", it should have been "children whose mothers had an unwanted…".
- The author used "1 day delay" as a delay. What is the basis of the 1 day? Personally, I would have consider 1h already as a delay, especially because it is question of children. The author should better argue the choice.

Conclusion:

I suggest the publication of this paper after consideration of the above propositions.
