# Supplementary material for: Delays in seeking treatment for fever in children under five years of age in Nigeria: Evidence from the National Demographic Health Survey
Source: PLoS One. 2023 Dec 20;18(12):e0295772. doi: 10.1371/journal.pone.0295772 (PMC10732371; doi:10.1371/journal.pone.0295772)
Supplement: Attachment — Submitted filename: Response to Reviewers.docx [file pone.0295772.s002.docx]

November 21^st^, 2023

Dr Sathiya Susuman Appunni

Academic Editor, PLOS ONE

**RE: Manuscript resubmission – [PONE-D-23-28224R1] Delays in seeking treatment for fever in children under five years of age in Nigeria: Evidence from the National Demographic Health Survey**

Dear Dr. Appunni,

Thank you for your constructive comments and those from the reviewers. A revised manuscript (clean and version with track changes) reflecting the point-by-point responses below have been submitted for your consideration. Note the modified tables (1, 2) are appended after the references in the manuscript.

Kind regards,

Dr. Anayochukwu Anyasodor, Ph.D.

E-mail: [aanyasodor@csu.edu.au](mailto:aanyasodor@csu.edu.au)

**Academic Editor**

**Comment:** Please ensure that your manuscript meets PLOS ONE's style requirements, including those for file naming. The PLOS ONE style templates can be found at <https://journals.plos.org/plosone/s/file?id=wjVg/PLOSOne_formatting_sample_main_body.pdf> and <https://journals.plos.org/plosone/s/file?id=ba62/PLOSOne_formatting_sample_title_authors_affiliations.pdf>

**Response:** The manuscript has been revised to meet PLOS ONE's style requirements.

**Comment:** Thank you for stating the following financial disclosure: "N/A. At this time, please address the following queries: a) Please clarify the sources of funding (financial or material support) for your study. List the grants or organizations that supported your study, including funding received from your institution. b) State what role the funders took in the study. If the funders had no role in your study, please state: “The funders had no role in study design, data collection and analysis, decision to publish, or preparation of the manuscript.” c) If any authors received a salary from any of your funders, please state which authors and which funders. d) If you did not receive any funding for this study, please state: “The authors received no specific funding for this work.”

Please include your amended statements within your cover letter; we will change the online submission form on your behalf.

**Response:** We have edited this in the revised manuscript (Page 15, line 303). The financial disclosure statement reads: *“**The authors received no specific funding for this work.”*

**Comment:** Thank you for stating the following in your Competing Interests section: "Authors have no conflict of interest to declare." Please complete your Competing Interests on the online submission form to state any Competing Interests. If you have no competing interests, please state "The authors have declared that no competing interests exist.", as detailed online in our guide for authors at <http://journals.plos.org/plosone/s/submit-now> This information should be included in your cover letter; we will change the online submission form on your behalf.

**Response:** We have edited this in the revised manuscript (Page 15, line 304). The competing interests statement reads: *“The authors have declared that no competing interests exist.”*

**Comment:** We note that [Figure 1] in your submission contain [map/satellite] images which may be copyrighted. All PLOS content is published under the Creative Commons Attribution License (CC BY 4.0), which means that the manuscript, images, and Supporting Information files will be freely available online, and any third party is permitted to access, download, copy, distribute, and use these materials in any way, even commercially, with proper attribution. For these reasons, we cannot publish previously copyrighted maps or satellite images created using proprietary data, such as Google software (Google Maps, Street View, and Earth). For more information, see our copyright guidelines: <http://journals.plos.org/plosone/s/licenses-and-copyright>. We require you to either (1) present written permission from the copyright holder to publish these figures specifically under the CC BY 4.0 license, or (2) remove the figures from your submission:

**Response:** We have now removed Figure 1 from the revised manuscript.

**Comment:** Please review your reference list to ensure that it is complete and correct. If you have cited papers that have been retracted, please include the rationale for doing so in the manuscript text, or remove these references and replace them with relevant current references. Any changes to the reference list should be mentioned in the rebuttal letter that accompanies your revised manuscript. If you need to cite a retracted article, indicate the article’s retracted status in the References list and also include a citation and full reference for the retraction notice.

**Response:** We have edited the reference in the revised manuscript.

**Reviewer #1**

**Comment:** The manuscript is technically sound, and the data support the study's conclusion. Moreover, the statistical analysis has been performed appropriately and rigorously. Furthermore, the authors made all data underlying the findings in their manuscript fully available. I found the English language editing in this article unambiguous.

**Response:** We thank the reviewer for the comments. The reviewer’s specific comments are addressed below in this rebuttal.

**Comment:** However, I suggest more reporting on bivariate analysis, where the study factors were cross-tabulated with the outcome variables in Table 1. The authors would have looked at the pattern of the percentages and reported the relationship because there is valuable information related to the topic under study.

**Response:** We have edited this in the revised manuscript (Table 1, appended after references).

**Comment:** On page 12 of the article, I was confused. Under the subheading: "Prevalence of delay in treatment seeking for fever," (The prevalence of delay in treatment-seeking for fever was 62.1% (95%CI: 60.1, 64.1%), which was higher in children whose mothers did not attain a formal education (69.1%), and those who resided in poor households (70.0%) [Table 1]). I need help finding where the authors used the Confidence Interval in Table 1. CI was only used under the multivariate section, where they used binary logistic regression to identify the factors contributing to the predisposing, enabling, need, services, and community factors.

**Response:** We have edited this in the revised manuscript (Page 11, lines 218-219)

**Comment:** As it is recommended by PLOS policy, the data should be provided as part of the manuscript or its supporting information for verification.

**Response:** We acknowledge the reviewer's concern and have addressed it by including a data-sharing statement in the revised manuscript (Page 15, lines 313-318): The data used for this study are available from The DHS Program. Users can register on the DHS Program website (<https://dhsprogram.com/>). Once registered, interested researchers can request access to the datasets. The 2018 Nigeria DHS data are available for download at the following link: <https://dhsprogram.com/data/dataset/Nigeria_Standard-DHS_2018.cfm?flag=0>. The authors confirm they had no special access privileges.

**Reviewer #2**

**Comment:** The authors should find a basis for the choices made to declare that 1 day is a delay and, therefore, before a day, it is not a delay.

**Response:** We selected 24hrs for defining a delay in seeking care for fever based on information from the Guide to DHS Statistics (page 12.24, available at <https://www.dhsprogram.com/pubs/pdf/DHSG1/Guide_to_DHS_Statistics_DHS-7_v2.pdf>) and previously published studies *(Bekele et al., 2023; Debsarma et al., 2022; Hamooya et al., 2016; Rutebemberwa et al., 2009)*. This is now reflected in the revised manuscript (Page 7 lines 142-145)

**Comment:** Very few recommendations came from the study. But, so many good results were obtained. It would help if you improved your recommendations.

**Response:** The ‘Conclusion’ has been revised (Page 14, lines 294-299).
